# Multicriteria Methodology for Prioritizing Predictive Maintenance Using RPASs (Drones) with Thermal Cameras on Transmission Lines

**DOI:** 10.3390/s25165064

**Published:** 2025-08-14

**Authors:** André Schnorr, Daniel Bernardon, Dion Feil, Francisco Fabrin, Cristiano Konrad, Laura Lisiane Callai dos Santos, Vagner Bitencourt, Herber Fontoura, Cristian Correa

**Affiliations:** 1 Headquartes Campus, Federal University of Santa Maria, Santa Maria 97105-900, RS, Brazil; dpbernardon@ufsm.br (D.B.); dion.feil@ufsm.br (D.F.); francisco.fabrin@acad.ufsm.br (F.F.); ckonrad@inf.ufsm.br (C.K.); laura.santos@ufsm.br (L.L.C.d.S.); 2 CPFL Energia, Campinas 130088-900, SP, Brazil; vagner.bitencourt@cpfl.com.br (V.B.); herber.fontoura@cpfl.com.br (H.F.); cristian.correa@cpfl.com.br (C.C.)

**Keywords:** RPAS, AHP, thermography, maintenance, prioritization, drones, transmission lines

## Abstract

Thermographic inspections using drones with thermographic cameras have enabled considerable advances in preventive maintenance. In this context, we propose a methodology for prioritizing flight performance to ensure that the equipment is used in the most efficient way, based on the implementation of thermographic technology in the preventive maintenance plans of electric power concessionaires. Based on information about transmission lines obtained from the literature and made available by transmission companies, criteria and alternatives are established, and a methodology for prioritization and application in transmission lines is established using the AHP multicriteria method. Technical, safety, systemic, social, and financial criteria are defined, each containing alternatives, to define their weight and importance. Finally, through analysis of the four established criteria and alternatives, with their respective weights, a tool is obtained that will assist transmission concessionaires in the adequate prioritization of thermographic inspections using RPAS.

## 1. Introduction

Electricity is essential for the harmonious functioning of all sectors of society. In the context of continuously increasing demand on the supply continuity and quality of energy delivered to consumers, interruptions, low energy quality, and supply security issues are incurring increasingly harsh penalties and fines according to standardized indicators [1].

Due to this increase in demand, energy concessionaires have developed a variety of methods to ensure increased reliability and quality in their delivery of electricity, achievements that are facilitated by technological advances.

Failures in the supply of electrical energy by electrical energy transmission companies, especially in high-voltage networks, are critical problems that have received increasing attention, considering the costs involved in the process and the problems caused by these defects for both for concessionaires and society in general.

With the emergence of new technologies and the improvement of others, it has become essential to improve the maintenance processes of electrical energy transmission concessionaires. Drones have evolved significantly in terms of their flight capability, onboard technology, and cost, making their use for maintenance on electrical networks increasingly viable and important, which was the main motivator for carrying out this work [2].

In the most diverse spheres of modern society, accurate and effective decision-making is a crucial element for the success of any given project. In the electrical energy sector in particular, taking into account electrical energy transmission systems, decisions are required in planning, expansion, operation, the location of improvements in the network, reinforcements, and resource allocation forecasts [3].

In this context, this work meets the need to develop optimized and robust preventive maintenance plans by integrating new thermographic inspection technologies using drones, ensuring optimized flights that are carried out at the best points for transmission line companies and guarantee satisfactory results based on their criteria and relevance.

There is a lack of research that covers all the areas proposed in this paper. There are several articles that partially address these topics, but none have been found that encompass all of them, demonstrating a need to develop new methodologies that assist in standardizing decision-making by leadership and engineering professionals in electricity transmission companies, reducing subjectivity in decision-making, and adequately improving maintenance plans, including integrating the most recent technologies.

Developing this methodology requires connected four major related areas, namely multi-criteria analysis (AHP); transmission lines; remotely piloted aircraft systems (RPASs), which are popularly known as drones; and thermography.

Publications [4,5,6,7,8,9,10] address techniques for carrying out preventive inspections on transmission lines using RPASs with thermal cameras, demonstrating the importance of using this technology and the impact of using aircraft to carry out inspections.

The first studies, especially [6,9], provide an important basis for better understanding the thermographic inspection technique using drones, also showing how image processing helps identify defects, which are delegated to maintenance teams to resolve.

Publications [11,12,13,14,15,16,17,18,19,20] report the use of the AHP method to prioritize and optimize processes in several areas. In [11,12,13], the method is used for thermography, but in different areas such as industry, closed-circuit television (CCTV) and polymer composites; however, these applications have proven to be very useful, as they elucidate the possibilities of applying AHP using the thermographic technique in various areas.

The authors of [14,15,16,17] describe the use of RPASs to clean solar panels, define the need to purchase a drone, in maritime installations, and for belief management in internet technology, respectively, applications that all used the AHP method to optimize and prioritize processes, sometimes in combination with other multi-criteria decision analysis (MCDA) techniques. These articles are important when it comes to applying AHP in drones, contributing to a better understanding of its applications and aiding in the creation of criteria and alternatives.

Finally, the authors of [18,19,20,21] apply AHP to transmission lines, mainly to defects; among these studies, [18,21] are most relevant to the search for prioritization and the establishment of criteria used for transmission lines. In [21], criticality matrices are demonstrated, which are widely used by energy transmission companies. These articles also elucidate several points to be considered in asset management and the prioritization of maintenance activities in transmission lines, which were very useful for the composition of this paper. 

This work proposes incorporating the use of thermographic drones into the routine maintenance tasks of an electricity transmission company and developing a methodology for prioritizing predictive maintenance activities in transmission lines through the use of a multi-criteria methodology for decision-making in the preparation of a preventive maintenance plan.

Thus, the main contributions of the work are as follows:

- Describing a new method for prioritizing predictive maintenance activities for transmission lines;
- Creating a tool for supporting decision making regarding the prioritization of inspections in transmission lines;
- Optimizing inspections using drones with thermographic cameras.

## 2. Materials and Methods—RPAS with Thermal Imaging Camera and Application of AHP

Drones with different thermographic camera configurations were used to prepare the technique and verify its limitations, with the aid of professionals specializing in thermography and drone pilots. Spreadsheets were used to administer questionnaires to the experts, and tables were prepared for data processing.

Thermography involves the collection and analysis of thermal data using imaging equipment that does not require physical contact [22]. Thermal detection is based on the physical law that all bodies radiate heat in the form of infrared radiation, the intensity of which is directly related to the temperature of the body. The device used to measure this thermal data, a thermal imager, records this infrared radiation and transforms it into a representation of temperature.

Thermography is used in a wide range of fields that require where non-invasive and non-destructive temperature measurement, especially when equipment reliability is essential and a problem needs to be detected and corrected before a failure occurs.

When discussing any part of an electrical system, it is essential to consider the properties of the materials involved in the process. One of the main factors influencing the choice of material used is how it behaves in relation to temperature variation when current is passed through it.

Problems in the conduction of electrical energy through the elements comprising an energy transmission system can result in excess heat, which can be identified using thermography. This is a very efficient predictive maintenance technique for detecting anomalies resulting from faulty contacts between power conduction circuits, with the ability to classify detected faults according to their temperature and location characteristics. This enables decision-makers to determine an appropriate time for corrective maintenance before system failure occurs.

In thermographic inspections on transmission lines, it is essential to understand which pieces of equipment along the line must be checked and evaluated:

- Contacts and connections;
- Preformed and compression joints;
- Insulators;
- Cover cables.

**Figure 1:**
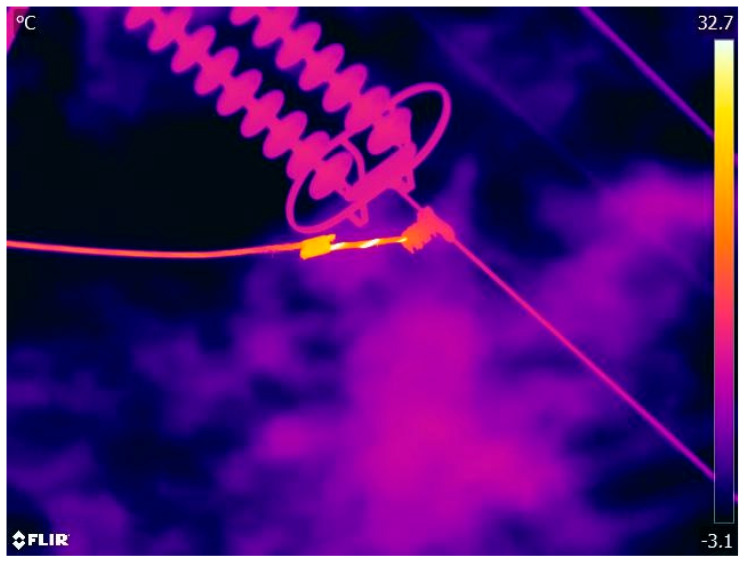
Thermographic image of thermal anomaly in transmission lines.

To understand the process of a thermographic inspection, regardless of how it is performed, it is important to understand the image processing conducted after the inspection is carried out to make temperature adjustments until the final value to be considered is found. An example of a processed image is shown in Figure 1. This image was taken with a thermographic camera on the ground and processed using the manufacturer’s software.

Aerial thermographic inspections are currently carried out using helicopters, with a thermographer performing the analysis from inside the aircraft, using handheld equipment. Transitioning to thermography using drones makes it easier to carry out inspections, since there is no need for an aircraft with a pilot; this also reduces the costs involved with the activity.

The word drone was first introduced to describe remotely piloted aircraft without the need for a person on board. It refers to “drone as bee male”, due to the characteristics of the first equipment, such as its sound and flight pattern.

When using technical terms to characterize unmanned aircraft, it is necessary to characterize each one according to its application and characteristics. They can generally be defined as Unmanned Aerial Vehicles (UAVs) and Remotely Piloted Aircraft Systems (RPASs), but in this study, the term drone is used for ease of communication.

It is important to know the type of piloting, according to the classification made in [23]. There are three main types: BVLOS (Beyond Visual Line-Of-Sight), where the pilot is unable to see the aircraft even with the support of an observer; VLOS (Visual Line-Of-Sight), where the pilot can have visual contact with the drone without the support of an observer or other equipment such as lenses or others; and EVLOS (Extended Visual Line-Of-Sight), where visual contact with the aircraft is maintained by observers or auxiliary equipment. It is also important to note that autonomous flight, i.e., unmanned aircraft without a pilot, is not authorized by Brazilian law [24].

The use of a drone with a thermographic camera enables better analysis of anomalies, since it replaces ground inspection and can bring the thermographic camera closer to the hotspot, reducing the influence of weather conditions and distance from the object and ensuring that the images captured better portray the conditions of the anomaly.

It is essential to understand the objective of aerial thermographic inspections of transmission lines since it should accompany a visual inspection, providing an additional tool to identify weak points in the transmission line that are not perceptible to the naked eye.

According to the authors of [25], with the variety of existing Multi-Criteria Decision Analysis (MCDA) methods, decision-makers are faced with the complex challenge of choosing an appropriate tool to support their decision, a choice that is often difficult to substantiate.

Multicriteria decision-making, specifically the AHP method, which was developed by Thomas Saaty in the 1970s, is a technique that helps decision-makers structure and prioritize problems [26].

The AHP method is based on the comparison of criteria (i.e., electrical and energy variables linked to indicators of quality and continuity of energy) in pairs, using a scale that correlates the importance of one criterion with that of another. Each criterion is assigned a degree of importance, established according to a numerical scale, as proposed in [27].

This work is guided by the objective of developing a methodology that incorporates practices already used by transmission and energy companies and a new tool available on the market. There are currently no prioritization methodologies for this purpose.

Several criteria and alternatives have been established in the literature, but [21] is the most relevant to energy transmission companies. This study offers a criticality matrix that considers the probability of the occurrence of defects in relation to the severity of defects in transmission lines.

In [21], the criticality analysis for maintenance management has four classifications, namely low, medium, high, and very high. The main criteria highlighted relate to efficiency, safety/reliability, environment, and finance, similar to what is currently practiced.

## 3. Methodology

The proposed methodology primarily comprises the use of multicriteria analysis to prioritize preventive maintenance activities for transmission lines, with a view to implementing drone technology in a concessionaire’s preventive maintenance plan, particularly drones with thermal imaging cameras attached.

This approach is based on a combination of works studied, mainly [6,11,19], and the current maintenance processes of several electricity transmission companies.

**Figure 2:**
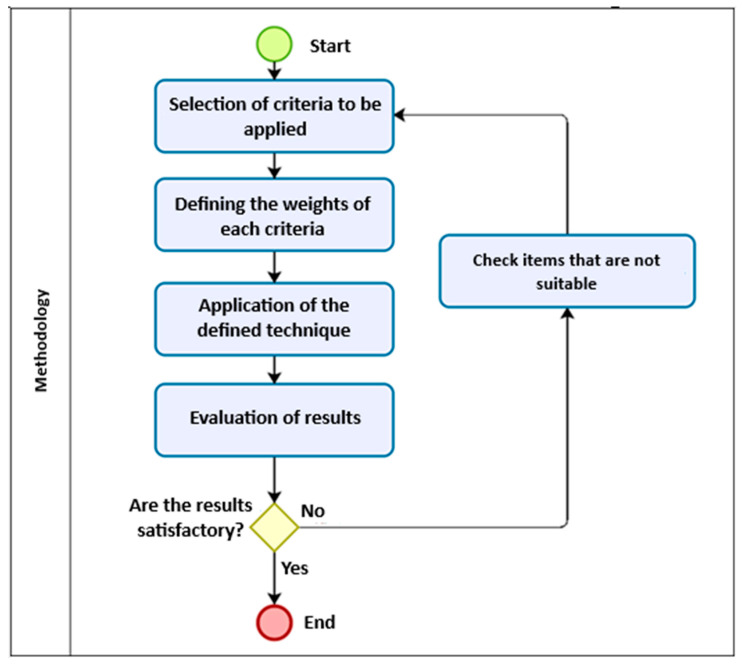
Flowchart of proposed methodology.

Figure 2 shows a detailed flowchart of the methodology, where the criteria to be adopted are first selected with their alternatives and their weights are defined, followed by the application of the technique through consultation with experts and use of the AHP method. Finally the results obtained in this work are evaluated in relation to the experts’ guidance, with necessary adjustments made to items that may not produce satisfactory results.

### 3.1. Step 1: Defining Criteria and Weights

At this stage, possible criteria are identified based on the bibliography presented, current practices, and the vast knowledge of professionals in the field, who will assist in the development of the project.

The selection of criteria and their divisions is the most significant part of the process for applying AHP, given the breadth of issues involving TL activities and the various consequences of a failure or supply interruption due to a defect.

The weights are defined according to the authors’ knowledge based on the current approach to managing the concessionaire’s assets, references researched, and courses and workshops held in the past, which provide a basis for adequately establishing the weights for each of the defined elements, which are subsequently validated by consulting experts in stage 2.

### 3.2. Step 2: Application of the Criteria to Transmission Lines and Validation of the Results

Stage 2 involves applying the technique developed using AHP to transmission lines, demonstrating the prioritization of inspections using drones with thermal imaging cameras based on the criteria and weights defined in stage 1; validation with experts, which involves engineers and managers; and making adjustments if necessary, ensuring the validity of the methodology and confirming the results of the work.

**Figure 3:**
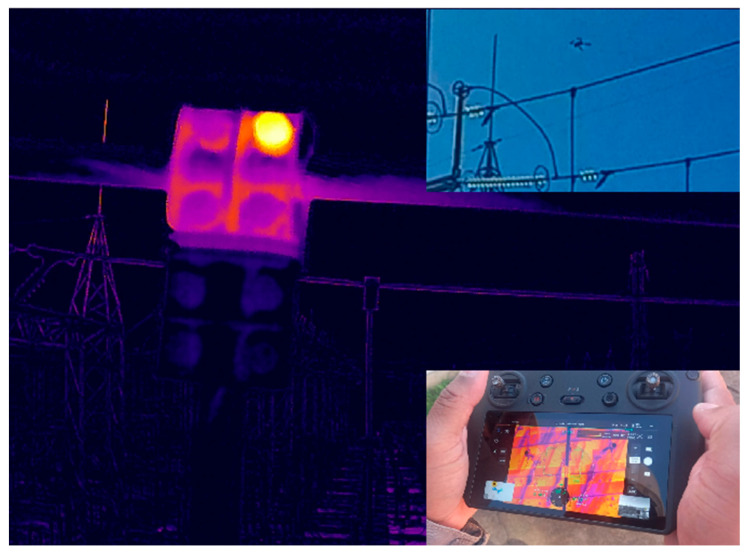
Thermal image of thermal anomaly captured by drone.

Several flights were performed using drones with thermographic cameras to ensure the robustness of the established criteria, as demonstrated by a thermographic image taken by a drone (Figure 3).

Since there is complexity in assigning weights in relation to the defined criteria, an AHP model is used for validation by professionals, with a view to equalizing the information and ensuring that resulting decisions are aligned with the company’s history of action for system occurrences, considering the reasons for events with the greatest repercussions and most resulting damage from technical and administrative perspectives.

In the first step of the methodology, the choice of macro processes for defining the criteria is essential to understanding the scope of the topic, considering the number of factors required to make the best decision. To this end, four main points are defined globally, based on the key criteria considered by electricity transmission companies: technical, safety, systemic/social, and financial criteria. These criteria are based on approaches currently implemented by some transmission concessionaires, with some points modified according to the expertise of the author, some professionals in the field, and the literature reviewed. Some adjustments were also made to ensure the criteria are specific to thermographic inspections carried out using drones. When defining the criteria, it is essential to understand two points:

- The classification is determined by any of the elements present in the criterion;
- The alternative with the highest weight will prevail over the others, with “0” being the most relevant and “3” being the least important.

### 3.3. Criterion 1—Technical

To present this criterion, information relevant to the history of the transmission line is observed, such as a general assessment of the defects present (criticality, quantity, ease of resolution), equipment operating time, history of trips, and approximate travel time of teams (including difficulty in reaching the equipment), among others.

Table 1 presents information based on practices in the electricity transmission sector and authors such as [21], showing the prioritization of defect care according to the risk ratio, management time required for care, the characteristics of the defect and, finally, the treatment required in relation to the behavior of the executive team.

The following alternatives are considered for this item:

T0—Equipment has a defect with Urgent or Emergency status or the Line has a history of shutdown due to a fault similar to the defect found;

T1—Has Necessary defects;

T2—Has Desirable defects;

T3—Has Manageable defects or no significant defects are recorded.

The main component to be evaluated in this criterion is the risk of the Transmission Line experiencing a failure or forced shutdown. This requires direct observation of related issues, among which the following are the most prominent:

- History of observed LT failures;
- History of failures due to defects observed in preventive maintenance; —Seasonality of the load;
- Location of the thermal anomaly (points at which there is a risk of a cable or connector breaking);
- Verification of the significance of the defects observed based on their criticality.

### 3.4. Criterion 2—Safety

This assessment is based on consultations with various energy transmission companies regarding the points of greatest risk to life, including items such as the presence of encroachments or intrusions, risk to the community, passage over important roads or roads that pose risks in the event of a defect, animal husbandry; in short, all factors that pose a risk to living beings.

It is important to understand that S0, S1 and S2 address a risk to life, but S3 relates to risks to places that may have historical, cultural, or religious value.

The following alternatives are considered for this item:

S0—Buildings in the right of way of the transmission line that serve as housing (encroachments or intrusions);

S1—Highways with high traffic that passes under the transmission line at at least one point;

S2—Lines where animals are raised in the right of way;

S3—Lines that do not fit into the other three options.

### 3.5. Criterion 3—Systemic/Social

Like the safety item, this assessment is based in the experience of transmission companies and defines the systemic impact of the possible shutdown of the Transmission Line, such as power interruptions, their impact on society (hospitals, trains, public safety agencies, and telecommunication centers), the number of people affected, and side effects of cascading shutdowns.

The following alternatives are considered for this item, as they are impacted by the shutdown of the transmission line:

SS0—Load shedding in hospitals, trains, public safety agencies, and essential service providers;

SS1—Load shedding occurs except for at SS0 locations;

SS2—Loss of the (*n*−1) criterion;

SS3—There is no direct impact on system configuration.

### 3.6. Criterion 4—Financial

The financial criterion assesses the impact resulting from fines, loss of revenue, and other penalties imposed on the transmission company due to unscheduled shutdowns that directly impact its income. The impact of layoffs on the company’s image and value must also be assessed here. This item considers the principal outcomes of financial losses in a generic way, considering that each country has its own regulations.

The following alternatives are considered for this item:

F0—Ranks among the 10 most profitable companies, has a high risk of negative impact on the company’s image, has a high risk of requiring an inspection process subject to fines, or has a high risk of inclusion in the resulting plan.

F1—Ranks among the 30 most profitable companies, has a considerable risk of negative impact on the company’s image, has a considerable risk of requiring an inspection process subject to fines, or has a considerable risk of inclusion in the resulting plan;

F2—Ranks below the 30 most profitable companies, has a medium risk of negative impact on the company’s image, has a medium risk of requiring an inspection process that could result in fines, or has a medium risk of inclusion in the resulting plan;

F3—Ranks below the 30 most profitable companies, has a low/non-existent risk of negative impact on the company’s image, has a low/non-existent risk of requiring an inspection process subject to fines, or has a low/non-existent risk of inclusion in the resulting plan.

### 3.7. Summary of Criteria

As presented above, the criteria are divided according to the areas to which they belong. Table 2 provides a summary of all criteria plus the established alternatives, which serve as a basis for assembling the decision matrices after inserting the weights for each item, which are defined following the consultation with experts.

### 3.8. Application of Proposed Technique and Evaluation of Results

Section 4 and Section 5 discuss the application of the proposed technique and the evaluation of the results to validate and ensure the correct application of the methodology, taking into account the steps already defined in this section.

## 4. AHP Application

The AHP (Analytic Hierarchy Process) is a discrete multicriteria method and important tool for Multi-Criteria Decision Analysis (MCDA). It was one of the first to be developed and has been widely adopted globally. This method was chosen based on its expected inputs and characteristics.

Given the subjectivity and diversity of the existing criteria and the necessary analyses, in addition to the possibility of comparison between pairs on a proportional scale, the AHP method proves suitable for developing the methodology proposed in this work, contemplating the experts’ evaluations in an appropriate manner, and adhering to the proposed method.

The AHP method is applied in the second stage of the proposed methodology, according to the selection of hierarchy and prioritization criteria chosen by means of an expert system—in this case, the parameters were defined following expert consultation, and have been demonstrated in the literature review to be validated by professionals with extensive experience with transmission lines.

Following the AHP method, the hierarchical decision levels were defined. They are shown in Table 3, which describes the problem, criteria, and alternatives.

**Figure 4:**
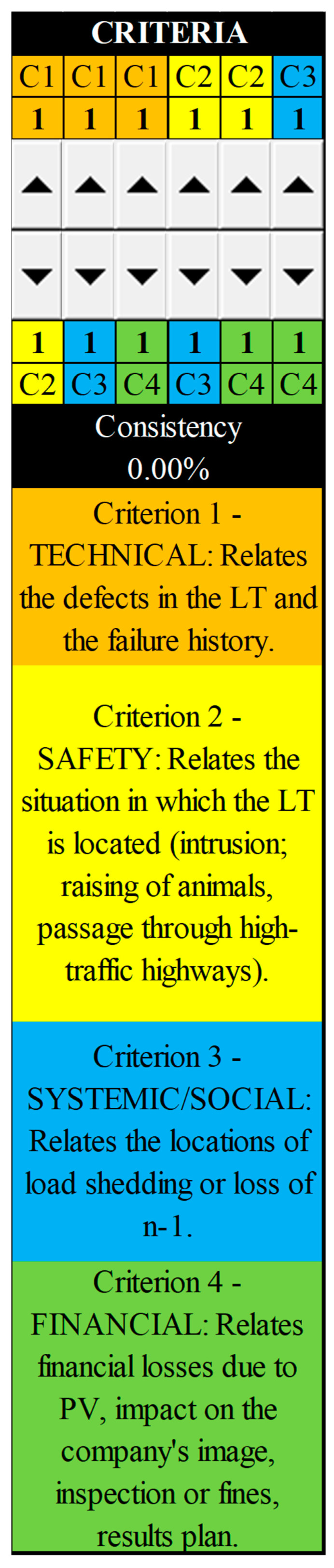
Part of questionnaire—criteria for weights.

A qualitative research questionnaire was prepared to define the values for each criterion adopted and the alternatives for each criterion. It was as a reference for calculation and subsequent validation by experts, as shown in Figure 4.

### 4.1. Application of AHP to the Criteria

The values were defined according to the authors’ expertise and structured based on the recent history of decision-making regarding the use of visual drones in CPFL transmission, considering that drones with thermal cameras are a very recent technology and are still being studied through R&D activities. These weights were validated later with other experts.

Defined criteria are used in different ways in the electricity sector, normally through matrices that relate the impact of an event (safety, systemic, social and financial) with its probability (technical).

In total, four criteria were defined, as shown in Table 4. Using the data contained therein, the consistency matrix was assembled, where a pairwise relative comparison was made for each of the criteria accounted for, as shown in Table 5. The weights determined after applying the calculations related to the AHP method are shown in Table 6.

The values contained in the matrix shown in Table 5 enable the determination of the auxiliary parameters responsible for verifying the consistency ratio (CR) indicator.

(1) $$CI=\frac{\lambda_{max}-n}{n-1}=\frac{4.0087-4}{3}=0.0029\;or\;0.29\%$$

(2) $$CR=\frac{CI}{RC}=\frac{0.0029}{0.9}=0.0032\;or\;0.32\%$$

where CI is the Consistency Index, RC is the Random Consistency index, λmax is the maximum eigenvalue of the consistency matrix, and *n* is the size of the matrix [26].

The resulting CR < 0.1 confirms the consistency of the hierarchy of the criteria listed above [26].

### 4.2. Application of AHP to Alternatives

Similar to the application of the criteria, the alternatives were applied, with the results expressed in Table 7. This table shows the percentages relative to each alternative and of the total, CI, and CR values for each one. It also ranks each alternative in relation to the others.

Adding the alternatives of greatest significance in each criterion indicates that the maximum percentage that a transmission line can reach is 61.84%. Finally, Table 8 shows the increasing ranking of the alternatives with their respective absolute percentages of importance for decision-making.

## 5. Results of Applying the Methodology

Professionals with different characteristics were consulted, to verify their understanding of the proposed criteria and alternatives, leaving room for suggestions of alterations, additions, and changes according to what the professionals understood should be present. The professionals’ characteristics are summarized in Table 9.

The professionals were chosen according to the unique insights their experiences afforded, which provided a variety of perspectives on the activity.

First, the AHP method was used for all the experts’ responses. A comparative analysis was made of each of the criteria defined in the work and already presented in the methodology, with the average result of each item of the experts’ questionnaire, which is presented in Table 10.

There is a notable difference in percentage points between the weights of the criteria, given that they are more subjective items, based on the professionals’ experiences and perceptions gained throughout their professional lives.

The percentage values of the alternatives are also shown in a comparative manner; these fluctuated much less in relation to the criteria in the comparisons, but when related to the final values, they were influenced by them. This had a significant impact on the final values, as shown in Table 11.

Finally, the ranking of the alternatives is demonstrated. The rankings are shown in a comparative way, demonstrating several changes; however, it is still possible to notice a general consistency in the position of the results, with the great influence provided by the difference in the values of the criteria. This is shown in Table 12, where Work Ranking is the ranking defined in this study, and Experts’ Ranking is the ranking defined following the consultation. In addition, the difference in positioning of each alternative is indicated in the Position Difference column.

## 6. Conclusions

This paper presents a case study on the optimization of predictive maintenance involving inspections using drones with attached thermal imaging cameras. Based on the research reviewed, the authors’ experience, and training provided, the criteria to be considered in the multicriteria selection method were defined, namely technical, financial, safety, and systemic/social criteria.

These criteria were introduced as input data into the multicriteria decision-making process. Using the AHP method, each criterion was ranked and assigned a weight, identifying the optimized solution for the most advantageous predictive maintenance plan that would meet the criteria proposed in this paper for the concessionaire’s transmission system. Due to the complexity of the problem, is the author recommends using a larger, more varied expert panel in future work. Although the sampling was quite consistent in this work, a larger sample could further improve the results.

It was possible to verify that the use of thermographic inspection using drones will be successful as long as it is directed appropriately. At this point, it is possible to understand the relevance and impact of the work both theoretically, considering that no studies with the same specificity were found, and practically, since it can serve as a basis for the development of an auxiliary tool for use by electric power transmission companies.

The results obtained align with expectations, with the initial research conducted with experts in the field proving to be extremely important. The disparity in responses from professionals directly involved with the activities to be carried out emphasizes the importance of standardization in decision-making.

The results of the scientific research and the results obtained through the literature review, in combination with the authors’ experience, proved to align closely with the way in which the experts defined their perceptions of the criteria, alternatives, and weight of each of the items evaluated, although there was some divergence in the results due to some subjectivity in the analyses, as previously mentioned.

Aligning with the objectives of this work, the presentation to company experts aims to create a standard for carrying out preventive maintenance after incorporating inspections using drones with thermal imaging cameras.

The aim of standardizing these inspections is to provide better service to support preventive maintenance, ensuring that the checks align with the company’s expectations and are not subject to individual analyses only, as individual analyses have been shown to be highly volatile, with a major impact on decision-making.

It is worth remembering that the results obtained should serve as a guide and may be adjusted according to regional characteristics or with the addition of items not listed in this work.

It was possible to verify that there is a high degree of complexity in standardizing preventive maintenance for transmission lines using drones with thermal imaging cameras. This complexity results from the number of particularities and different conditions to which the equipment is subjected, including geographic and meteorological conditions.

Despite this, the methodology was initially successful, with a view to reducing equipment failures and avoiding unscheduled shutdowns of transmission lines where the most critical criteria that were defined were present. It is also possible to note that the experts’ perceptions were noticeably different according to their technical and sectoral characteristics and alignment with the history of the company and management. These are important points to consider when broadly analyzing and evaluating the results and can, in fact, serve as a basis for making the type of decisions that constitute this work.

## Figures and Tables

**Table 1:** Prioritization of defects in transmission lines.

Priority	Risk	Manageable	Characteristics	Treatment
Manageable (maximum 3 years or up to 10 years)	Eventual or None	Yes, long term	Extendable	Monitors and schedules in a timely manner
Desirable (maximum 180 days or maximum 1 year)	Medium/low	Yes, medium term	Programmable	Program
Necessary (maximum 60 days)	High to equipment or third parties	Yes, short term	By necessity	Completes what is being done and meets
Urgency (maximum 15 days)	Imminent to equipment or third parties	No	Imposing	For everything and meets

**Table 2:** Prioritization of defects in transmission lines. Summary table of criteria with alternatives.

**1. Technical**				
**Type**	**Defect**	**Failure History**		
T0	Urgency/ Emergency	Analogous/General defect		
T1	Necessary	Analogous/General defect		
T2	Desirable	Analogous/General defect		
T3	Manageable	No significant defects		
2. Safety				
**Type**	**Situation**			
S0	Encroachment on right of way			
S1	Passes through high-traffic highways			
S2	Animals raised in right o way			
S3	Does not fit options 0, 1, or 2			
3. Systemic/Social				
**Type**	**Systemic conditions/Locations of load shedding**			
SS0	Load shedding—Hospitals, trains, public safety and essential agencies			
SS1	Load shedding—Other locations not classified as SS0			
SS2	Loss of *n*−1			
SS3	No load shedding or impact or irrelevant			
4. Financial				
**Type**	**Profitability ranking**	**Impact on the company’s image**	**Inspection**	**Resulting plan**
F0	Up to 10th position	High risk	High risk	Belongs/High risk
F1	Up to 30th position	Considerable risk	Considerable risk	Considerable risk
F2	Above 30th position	Medium risk	Medium risk	Medium risk
F3	Above 30th position	Low/ non-existent risk	Low/ non-existent risk	Low/ non-existent risk

**Table 3:** Prioritization of defects in transmission lines Hierarchical levels of decision-making.

Levels	Description
Level 1: Problem	What is the priority order for sending a drone with a thermal imaging camera to inspect transmission lines?
Level 2: Criteria	Technical–Safety–Systemic/Social–Financial
Level 3: Alternatives	T0 to T3-S0 to S3-SS0 to SS3-F0 to F3

**Table 4:** Criteria to be used in AHP method.

Criterion	Variable
1	Technical
2	Safety
3	Systemic/Social
4	Financial

**Table 5:** Consistency matrix of criteria to be evaluated by AHP [26].

	Crit 1	Crit 2	Crit 3	Crit 4
Crit 1	1	7	3	3
Crit 2	0.143	1	0.333	0.333
Crit 3	0.333	3	1	1
Crit 4	0.333	3	1	1

**Table 6:** Weights of variables defined by AHP method.

Crit	Weight
1	54.44%
2	6.88%
3	19.34%
4	19.34%

**Table 7:** Percentages for each alternative with CI and CR.

**Technicians**	**Percentages** **Normalized**	**Percentages Relative to the Total**	**Rank**
T0	60.24%	32.79%	1
T1	24.33%	13.24%	2
T2	10.46%	5.69%	5
T3	4.98%	2.71%	9
CI	2.55%		
CR	2.83%		
**Safety**	**Percentages** **Normalized**	**Percentages relative to the total**	**Rank**
S0	64.70%	4.45%	6
S1	14.81%	1.02%	14
S2	14.81%	1.02%	14
S3	5.67%	0.39%	16
CI	1.27%		
CR	1.42%		
**Systemic/Social**	**Percentages** **Normalized**	**Percentages relative to the total**	**Rank**
SS0	64.74%	12.52%	3
SS1	19.52%	3.77%	7
SS2	10.05%	1.94%	11
SS3	5.69%	1.10%	13
CI	1.37%		
CR	1.52%		
**Financial**	**Percentages** **Normalized**	**Percentages relative to the total**	**Rank**
F0	62.42%	12.07%	4
F1	19.33%	3.74%	8
F2	11.29%	2.18%	10
F3	6.96%	1.35%	12
CI	1.70%		
CR	1.89%		

**Table 8:** Ranking of alternatives with the highest absolute percentage.

Alternative	Percentages Relative to the Total	Ranking
T0	32.79%	1
T1	13.24%	2
SS0	12.52%	3
F0	12.07%	4
T2	5.69%	5
S0	4.45%	6
SS1	3.77%	7
F1	3.74%	8
T3	2.71%	9
F2	2.18%	10
SS2	1.94%	11
F3	1.35%	12
SS3	1.10%	13
S1	1.02%	14
S2	1.02%	14
S3	0.39%	16

**Table 9:** Characteristics of professionals consulted.

		Thermographer	Management Experience	Professional of Transmission Lines	Expert for over 5 Years
Specialist 1	Senior Electrical engineer	YES	YES	NO	YES
Specialist 2	Electrical engineer	NO	YES	YES	NO
Specialist 3	Senior Electrical Technician	YES	NO	NO	YES
Specialist 4	Electrical engineer	NO	YES	NO	YES
Specialist 5	Electrical engineer	YES	YES	YES	YES
Specialist 6	Electrical engineer Specialist	YES	YES	YES	YES
Specialist 7	Senior Electrical engineer	NO	NO	YES	YES
Specialist 8	Electrical engineer	NO	YES	YES	YES

**Table 10:** Comparative percentages of criteria.

Criticality	Work	Experts	Difference
Technical	54.44%	35.03%	−19.41pp
Safety	6.88%	16.41%	+9.53pp
Systemic/Social	19.34%	18.21%	−1.13pp
Financial	19.34%	30.35%	+11.02pp
CI	0.29%	2.40%	+2.11pp
CR	0.32%	2.67%	+2.35pp

**Table 11:** Comparative percentages of alternatives.

**Technicians**	**Percentages Normalized**			**Percentages Relative to the Total**		
	**Work**	**Experts**	**Difference**	**Work**	**Experts**	**Difference**
T0	60.24%	62.01%	+1.77pp	32.79%	21.84%	−10.96pp
T1	24.33%	21.09%	−3.24pp	13.24%	7.18%	−6.06pp
T2	10.46%	10.39%	−0.07pp	5.69%	3.71%	−1.98pp
T3	4.98%	6.51%	+1.54pp	2.71%	2.30%	−0.41pp
CI	2.55%	3.76%	+1.21pp			
CR	2.83%	4.18%	+1.35pp			
**Safety**	**Percentages normalized**			**Percentages relative to the total**		
	Work	Experts	Difference	Work	Experts	Difference
S0	64.70%	52.80%	−11.90pp	4.45%	7.93%	+3.47pp
S1	14.81%	27.33%	+12.51pp	1.02%	5.33%	+4.31pp
S2	14.81%	13.85%	−0.97pp	1.02%	2.22%	+1.20pp
S3	5.67%	6.03%	+0.36pp	0.39%	0.94%	+0.55pp
CI	1.27%	3.53%	+2.26pp			
CR	1.42%	3.92%	2.51pp			
**Systemic/Social**	**Percentages normalized**			**Percentages relative to the total**		
	Work	Experts	Difference	Work	Experts	Difference
SS0	64.74%	64.72%	−0.03pp	12.52%	12.03%	−0.49pp
SS1	19.52%	18.59%	−0.93pp	3.77%	3.13%	−0.64pp
SS2	10.05%	10.59%	+0.53pp	1.94%	1.93%	−0.02pp
SS3	5.69%	6.11%	+0.42pp	1.10%	1.12%	+0.02pp
CI	1.37%	3.30%	+1.93pp			
CR	1.52%	3.67%	+2.15pp			
**Financial**	**Percentages normalized**			**Percentages relative to the total**		
	Work	Experts	Difference	Work	Experts	Difference
F0	62.42%	65.37%	+2.94pp	12.07%	19.61%	+7.54pp
F1	19.33%	17.78%	−1.55pp	3.74%	5.50%	+1.77pp
F2	11.29%	10.20%	−1.09pp	2.18%	3.12%	+0.94pp
F3	6.96%	6.66%	−0.30pp	1.35%	2.12%	+0.77pp
CI	1.70%	3.23%	+1.53pp			
CR	1.89%	3.59%	+1.70pp			

**Table 12:** Comparative ranking of alternatives with the most influential criteria.

Alternatives	Work Ranking	Experts’ Ranking	Position Difference
T0	1	1	0
T1	2	5	−3
SS0	3	3	0
F0	4	2	+2
T2	5	8	−3
S0	6	4	+2
SS1	7	9	−2
F1	8	6	+2
T3	9	11	−2
F2	10	10	0
SS2	11	14	−3
F3	12	13	−1
SS3	13	15	−2
S1	14	7	+7
S2	14	12	+2
S3	16	16	0

## Data Availability

The original contributions presented in this study are included in the article. Further inquiries can be directed to the corresponding author(s).
